# Biphasic changes in β-cell mass around parturition are accompanied by increased serotonin production

**DOI:** 10.1038/s41598-020-61850-1

**Published:** 2020-03-18

**Authors:** Masaya Takahashi, Takeshi Miyatsuka, Luka Suzuki, Sho Osonoi, Miwa Himuro, Masaki Miura, Takehiro Katahira, Yuka Wakabayashi, Ayako Fukunaka, Yuya Nishida, Yoshio Fujitani, Satoru Takeda, Hiroki Mizukami, Atsuo Itakura, Hirotaka Watada

**Affiliations:** 1 Department of Metabolism and Endocrinology, Juntendo University Graduate School of Medicine, Tokyo, Japan; 2 Departments of Obstetrics and Gynecology, Juntendo University Graduate School of Medicine, Tokyo, Japan; 3 Departments of Pathology and Molecular Medicine, Hirosaki University Graduate School of Medicine, Hirosaki, Japan; 4 Laboratory of Developmental Biology & Metabolism, Institute for Molecular & Cellular Regulation, Gunma University, Maebashi, Japan

**Keywords:** Cell proliferation, Gestational diabetes

## Abstract

Pancreatic β-cell mass is known to be considerably altered during pregnancy and after parturition in rodents and humans. While β-cell mass increases during pregnancy and starts to return toward its original level after parturition, the cellular mechanisms by which β-cell mass during this period is regulated remains unclear. To address this issue in mice, we quantified β-cell mass and investigated the mechanisms underlying its regulation throughout the perinatal and postpartum period. The increased β-cell size and proliferation during pregnancy were significantly reduced shortly after parturition, whereas there was no evidence of β-cell reprogramming or increased apoptosis. Direct RNA sequencing of islets from pregnant and postpartum mice demonstrated dynamic changes in gene expression patterns, showing robust downregulation of cell cycle-related genes 1 day after parturition, and the reupregulation of serotonin metabolism-related genes at postpartum day 7. Serotonin synthesis was activated only in lactating females, accompanied by increased β-cell mass. Taken together, these findings demonstrate that β-cell mass is decreased shortly after parturition owing to reduced β-cell size and proliferation, and is subsequently increased, in association with lactation and serotonin biosynthesis.

## Introduction

Pancreatic β cells maintain their mass through a dynamic balance of proliferation, cell size, apoptosis, or transition to other cell types. During pregnancy, increased insulin resistance in the mother promotes nutrient transfer to the fetus through blood flow, whereas maternal β-cell expansion is induced by prolactin (PRL) and placental lactogen to compensate for the increased insulin resistance^1–3^. After parturition, maternal β-cell mass is known to return toward non-pregnant levels^4,5^, although the underlying molecular mechanisms involved remain unclear.

Transcriptome approaches have demonstrated the dynamic changes in gene expression profiles during pregnancy^6,7^. Among the genes most profoundly increased during pregnancy were *Tph1* and *Tph2*, which encode the two isoforms of tryptophan hydroxylase, the rate-limiting enzyme in serotonin (5-hydroxytryptamine, 5-HT) biosynthesis. Serotonin has been further shown to play a key role in β-cell proliferation and insulin secretion^7,8^. On the other hand, an *in vivo* study in Foxo1-deficient mice demonstrated that pancreatic β cells can be dedifferentiated after multiparity, reverting to a progenitor-like state^9^, implying that β-cell fate can be altered after parturition under certain specific conditions.

Thus, β-cell homeostasis is substantially altered during pregnancy and after parturition, although it remains to be elucidated as to when and how β-cell mass is decreased after parturition. To address these questions, we examined the temporal changes in β-cell homeostasis throughout the perinatal period, by investigating β-cell size, proliferation, and its cell fate, which demonstrated that reduced β-cell mass after parturition is determined by reduced cell size and proliferation rather than cell death or reprogramming. Furthermore, direct RNA-sequencing data from the islets of pregnant and postpartum female mice revealed the dynamic changes in mRNA profiles during the perinatal periods, showing robust downregulation of cell cycle-related genes shortly after parturition and re-upregulation of serotonin metabolism-related genes at postpartum day 7. Interestingly, serotonin production is activated only in lactating females, accompanied by increased β-cell mass, suggesting that lactation plays a role in expanding β-cell mass after parturition as well as placental lactogen during pregnancy. Understanding and controlling this process during the perinatal period may ultimately provide us with novel methods for expanding β-cell mass to treat diabetes mellitus.

## Results

### Temporal decrease and recovery of β-cell mass after parturition

**Figure 1:**
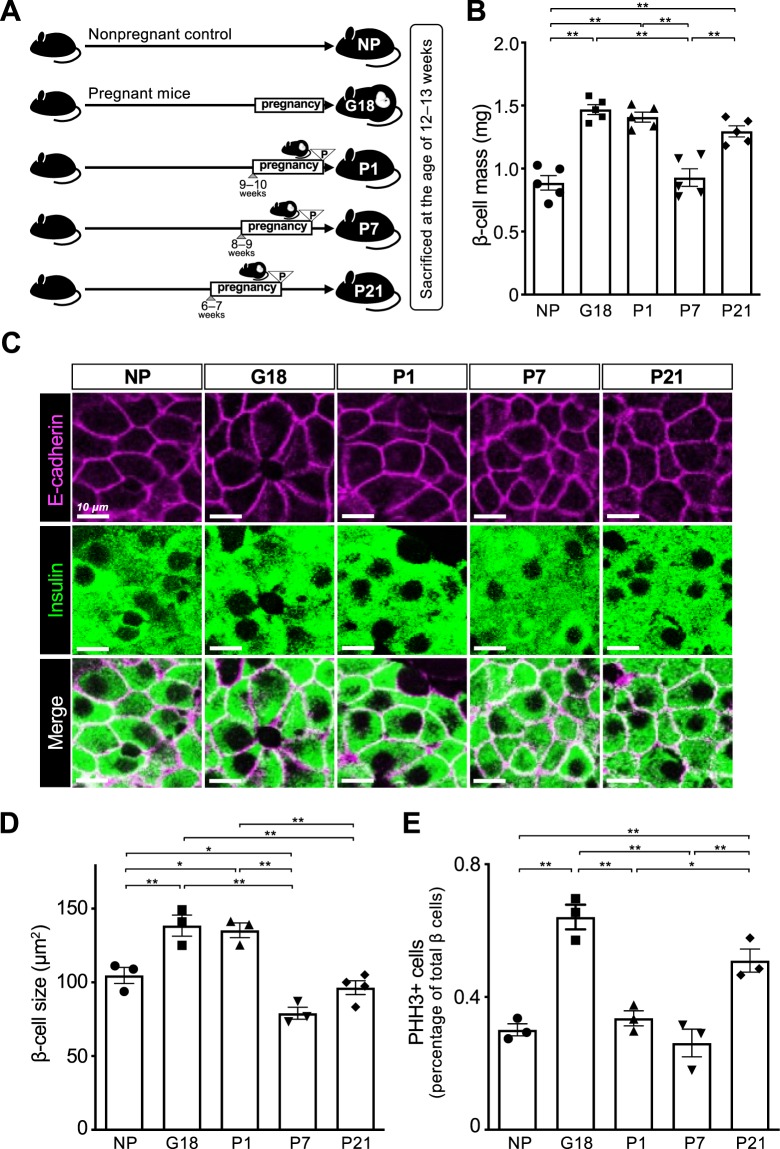
Temporal dynamics of β-cell mass, cell size, and proliferation during the perinatal period. (**A**) Schematic experimental design for the assessment of β-cell volume, β-cell size, and proliferation during the perinatal period. *C57BL/6 J* mice were sacrificed at different stages of perinatal stages (NP, G18, P1, P7, and P21) at the age of 12–13 weeks. The P-symbol indicates the day of parturition. (**B**) Quantification of β-cell mass (n = 5). (**C**,**D**) β-cell size was assessed through immunostaining for insulin (green) and E-cadherin (magenta) (**C**), and represented as bar graph with dots indicating individual data (n = 3–4) (**D**). The detailed methods are shown in Fig. S1. Scale bars, 10 μm. (**E**) The percentage of β cells positive for phosphorylated histone H3 (PHH3) out of a total number of more than 2,000 insulin-expressing cells (n = 3). All female mice that were sacrificed at P7 and P21 had breastfed more than 6 pups. Data are presented as the mean ± SE. NP, nonpregnant females; G, gestational day; P, postpartum day. *P < 0.05 and **P < 0.01.

To investigate temporal changes in total β-cell volume during the perinatal period, β-cell mass was quantified in non-pregnant females (NP), pregnant females at gestational day 18 (G18), and females on postpartum days 1, 7, and 21 (P1, P7, and P21, respectively) in mice on a C57BL/6 J background at 12–13 weeks of age (Fig. 1A). Immunostaining with an anti-insulin antibody and digital image analysis demonstrated that β-cell mass was significantly increased in pregnant females at G18, compared with non-pregnant females. Whereas β-cell mass at P1 was comparable to that at G18, it was significantly decreased at P7. Intriguingly, reduced β-cell mass at P7 was increased again at P21, which was significantly higher than that of NP (Fig. 1B).

### Reduced β-cell mass after parturition is determined by decreased β-cell size and proliferation rather than increased apoptosis

To clarify the cellular mechanisms by which β-cell mass is decreased at P7, possible mechanisms, such as β-cell size, proliferation, and apoptosis were assessed. Coimmunostaining for insulin and E-cadherin, which enabled us to precisely measure individual β-cell sizes (Figs. 1C and S1), demonstrated that the mean β-cell size was significantly increased during the perinatal period (at G18 and P1) compared with the nonpregnant period, and was robustly decreased at P7 and P21 (Fig. 1C,D). In addition, the percentage of cells expressing phosphohistone H3 (PHH3), a mitosis marker that is closely associated with mitotic chromatin condensation, was increased by almost twofold in pregnant mice at G18 compared with nonpregnant mice, robustly decreased after parturition at P1 to a level comparable to that of the nonpregnant period, and significantly increased again at P21, compared with NP or P7 (Fig. 1E). These findings suggest that the parturition event itself and/or subsequent changes, within a short period of time between G18 and P1, are associated with decreased β-cell proliferation. On the other hand, there was no significant difference between the groups in the number of terminal deoxynucleotidyl transferase-mediated dUTP nick-end labeling (TUNEL)-positive cells in the islets (Figs. S3A and S3B). Collectively, these findings suggest that the reduced β-cell mass after parturition resulted from a decrease in β-cell size and proliferation, rather than increased apoptosis.

### No evidence of β-to-non-β reprogramming after parturition

**Figure 2:**
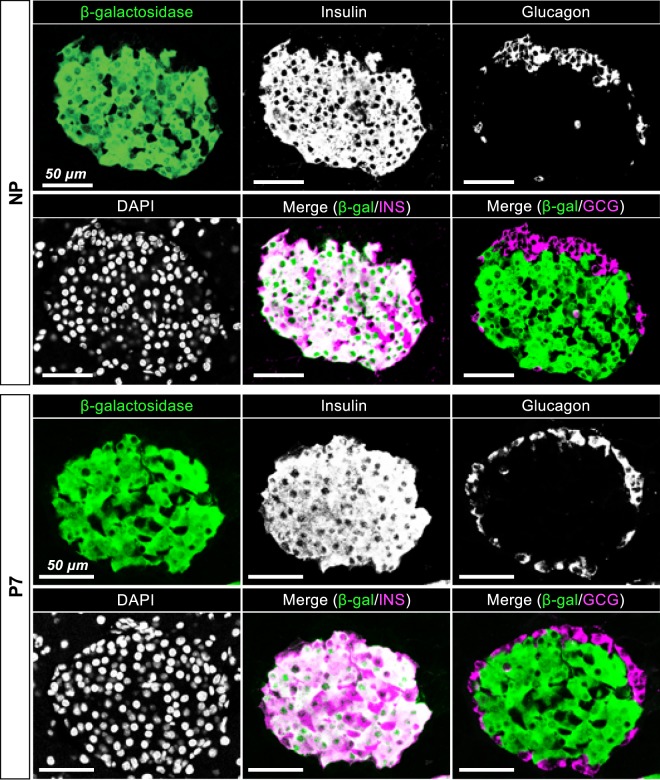
Lineage tracing of β-cell fate during the perinatal period. The β-cell lineage was traced in *Ins1-Cre*; *Rosa26*^*lacZ*^ (*βLacZ*) reporter mice at the age of 14–16 weeks. Immunostaining for β-galactosidase, insulin, and glucagon was performed in the pancreata of nonpregnant (NP) *βLacZ* mice and postpartum *βLacZ* mice at postpartum day 7 (P7). Nuclei were labeled with DAPI. Scale bars, 50 μm.

To further investigate whether β-cell dedifferentiation or transition towards non-β-cells contributes to reduced β-cell mass after parturition, the β-cell lineage was traced using *Ins1-Cre*; *Rosa26*^*lacZ*^ (*βLacZ*) reporter mice, in which insulin-producing β cells and their descendant cells were labeled as lacZ-expressing cells after Cre-mediated recombination. Fluorescent immunostaining for β-galactosidase (β-gal), insulin, and glucagon in the pancreata of *βLacZ* mice at NP and P7 demonstrated that all β-gal-expressing cells were positive for insulin and negative for glucagon, although there were a few insulin-expressing cells without β-gal expression, which had escaped Cre-mediated recombination. Taken together, these findings demonstrate that there is no evidence for the cellular transition of β cells to non-β cells during the perinatal period (Fig. 2).

### Temporal changes in transcriptome profiles demonstrated by RNA sequencing in the islets during the perinatal period

**Figure 3:**
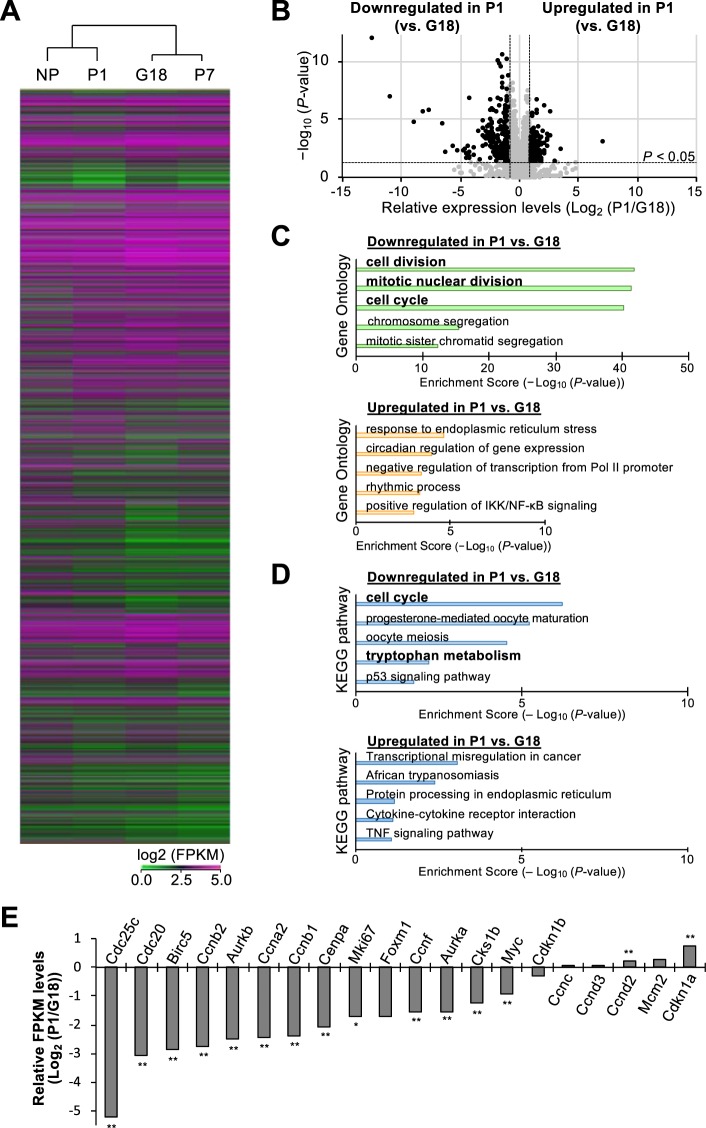
Transcriptome analysis of the islets during the perinatal period. Pancreatic islets were isolated from 12-week-old *C57BL/6 J* female mice at different perinatal stages (NP, G18, P1, and P7), and RNA sequencing (RNA-seq) was performed (*n* = 5–6 mice). (**A**) Overview of hierarchical clustering of RNA sequencing datasets in perinatal islets. A total of 11,826 genes are listed on the vertical axis. (**B**) Volcano plot comparing RNA-sequencing data from postpartum P1 females and pregnant females. Two dashed vertical lines represent the thresholds of log_2_ (Fold Change) (≧1 or ≦−1). (**C**) Gene ontology enrichment analysis was performed for the genes that are significantly downregulated in P1 compared with in G18. Top 5 gene ontology terms associated with biological processes are listed. (**D**) KEGG pathway enrichment analysis was performed for the genes that are significantly downregulated or upregulated in the islets of postpartum females at P1 compared with the pregnant females at G18. Top 5 KEGG pathway terms associated with biological processes are listed. (**E**) Relative expression levels of cell cycle-related genes in perinatal β cells. Changes in mRNA expression of selected cell cycle-related genes are presented as relative fold changes of mean FPKM between G18 and P1. NP, non-pregnant females; G, gestational day; P, postpartum day. *P < 0.05; **P < 0.01.

The findings described above demonstrate that the temporal dynamics of β-cell mass during the perinatal period are determined by a decrease in β-cell size and proliferation. To further investigate the molecular mechanisms underlying β-cell homeostasis during the perinatal period, transcriptome analysis by direct RNA sequencing (RNA-seq) was performed on islets isolated from NP, G18, P1, and P7 female mice. Hierarchical clustering and heatmap analyses demonstrated the dynamic changes in gene expression patterns in the islets during the perinatal period (Fig. 3A). Notably, unbiased clustering revealed similarities between NP and P1 as well as difference between G18 and P1, suggesting that global expression profiles in pregnant islets returned close to that of the non-pregnant state within a day after parturition. It is to be noted that there is more similarity between G18 and P7 rather than between the 2 postpartum days (P1 and P7), and this point is further analyzed and described later in this study.

### Dynamic changes in the expression levels of cell cycle-related genes after parturition

To further investigate the difference in mRNA profiles between G18 and P1, a volcano plot was constructed displaying statistical significance on the y-axis and fold change on the x-axis. As shown in Fig. 3B, 157 and 208 genes expressed in P1 are significantly upregulated and downregulated, respectively. Gene ontology (GO) analysis revealed that the many of the genes downregulated in P1 are genes that are involved in processes, such as “cell division”, “mitotic nuclear division”, and “cell cycle” (Fig. 3C), indicating that postpartum islets at P1 contain less proliferative cells compared with pregnant islets at G18. Likewise, Kyoto Encyclopedia of Genes and Genomes (KEGG) pathway analysis demonstrated that “cell cycle” was the most significantly downregulated pathway in the postpartum islets at P1 (Fig. 3D). In fact, the expression levels of positive regulators of cell division, such as *Cdc25c* and *Cdc20*, were robustly decreased in the islets at P1 compared with the islets at G18, whereas the expression level of the negative regulator *Cdkn1a* was significantly increased at P1 (Fig. 3E). It is noteworthy that *Cdkn1a* was significantly reduced in G18 islets compared with nonpregnant islets, which is consistent with the significant downregulation of *Cdkn1a* at midgestation (G13–G15) that we observed in our previous study^7^, whereas these positive regulators of cell division were not significantly increased in G18 islets compared with nonpregnant islets. These findings suggest that *Cdkn1a* plays an important role in regulating β-cell proliferation throughout gestation and parturition.

**Figure 4:**
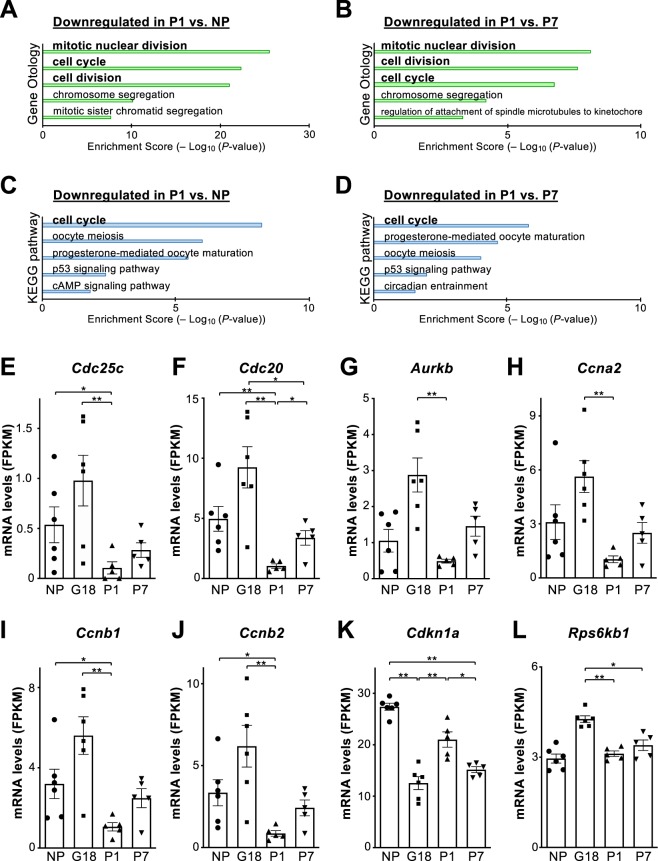
Dynamic changes in the expression levels of cell cycle-related genes during the perinatal period. (**A–D**) Gene ontology and KEGG pathway enrichment analyses were performed for the genes that are significantly downregulated in the islets of postpartum mice at P1, compared with the islets from nonpregnant mice (**A**,**C**) or postpartum islets from P7 mice (**B**,**D**). Top 5 gene ontology terms associated with biological processes or KEGG pathway terms are listed. (**E–L**) Expression levels (FPKM) of *Cdc25c* (**E**), *Cdc20* (**F**), *Aurkb* (**G**), *Ccna2* (**H**), *Ccnb1* (**I**), *Ccnb2* (**J**), *Cdkn1a* (**K**)*, and Rps6kb1* (**L**) are shown as bar graphs with dots indicating individual data (n = 5–6 mice), based on RNA-sequencing results. NP, non-pregnant females; G, gestational day; P, postpartum day. Data are presented as the mean ± SE. *P < 0.05 and **P < 0.01.

Interestingly, GO and KEGG pathway analyses between P1 and NP, or between P1 and P7, demonstrated the terms and pathways associated with cellular proliferation were enriched in the postpartum islets at P1 compared with islets from nonpregnant and P7 mice (Fig. 4A–J). In fact, the expression levels of positive regulators of cell division, such as *Cdc25c*, *Cdc20*, *Ccnb1*, and *Ccnb2*, were significantly decreased in the islets at P1, compared with islets not only at G18 but also from nonpregnant mice (Fig. 4E,F,I,J). In addition, a significant increase in *Cdc20* expression and a significant decrease in *Cdkn1a* expression were observed in the islets at P7 (Fig. 4F,K). Collectively, these findings suggest that cellular proliferation was robustly suppressed between G18 and P1, that is, within 48 hours around parturition, which may lead to a significant decrease in β-cell mass at later stages.

On the other hand, the expression levels of *S6K1* (*Rps6kb1*), which is known to regulate β-cell size^10^, were significantly decreased after parturition compared with during pregnancy (Fig. 4L), which may contribute to the decreased β-cell size after parturition (Fig. 1D).

### Enhanced serotonin signaling in islets of postpartum mice at P7 as well as pregnant mice

**Figure 5:**
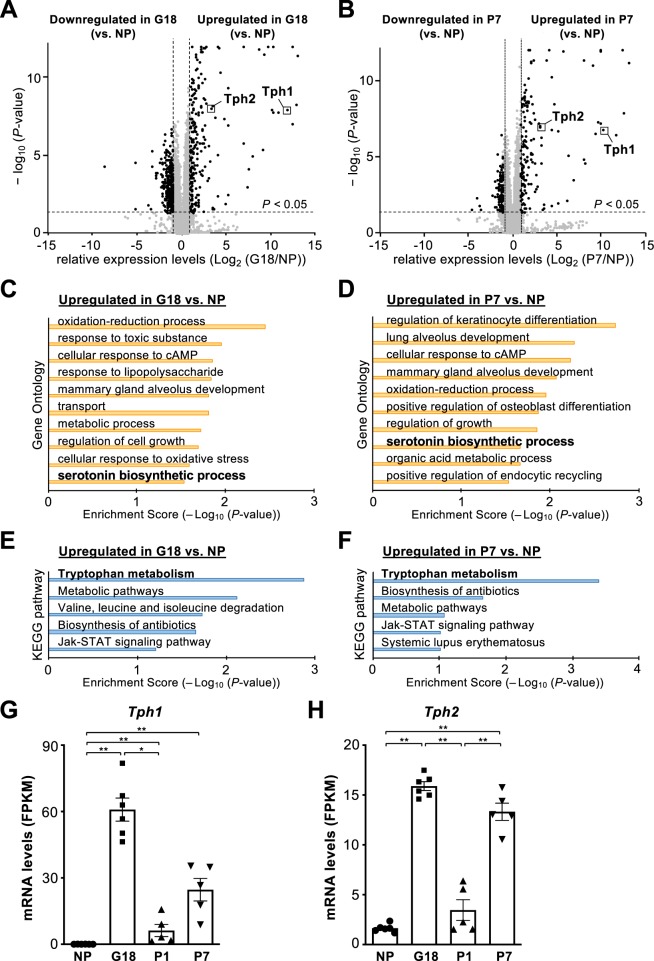
Dynamic changes in expression levels of serotonin metabolism- related genes during the perinatal period. (**A**,**B**) Volcano plots of RNA-sequencing data from pregnant female mice at G18 (**A**) or postpartum female mice at P7 (**B**), compared with non-pregnant female mice. (**C–F**) Gene ontology and KEGG pathway enrichment analyses were performed for the genes that are significantly upregulated in the islets of pregnant mice at G18 (**C**,**E**) or the postpartum mice at P7 (**D**,**F**), compared with the islets of nonpregnant mice. (**G**,**H**) Expression levels (FPKM) of *Tph1* (**G**) and *Tph2* (**H**) are shown as bar graphs with dots indicating individual data (n = 5–6 mice), based on RNA-sequencing results. NP, nonpregnant females; G, gestational day; P, postpartum day. Data are presented as the mean ± SE. *P < 0.05; **P < 0.01.

Gene expression profiles of cell cycle-related genes at P7 suggests the gradual recovery from robust suppression of cellular proliferation at P1. In addition, unbiased hierarchical clustering analysis demonstrated some similarity between pregnant islets at G18 and the postpartum islets at P7 (Fig. 3A). Therefore, we further investigated which genes are highly and commonly expressed in the islets at G18 and P7 compared with the nonpregnant islets, by constructing volcano plots of G18 vs. NP (Fig. 5A) and of P7 vs. NP (Fig. 5B), and through GO and KEGG pathway analyses (Fig. 5C–F). For 221 and 160 genes that were upregulated at G18 and P7, respectively (Fig. 5A,B), GO analysis demonstrated that the term “serotonin biosynthetic process” was significantly enriched in the islets at G18 and P7, compared with nonpregnant islets (Fig. 5C,D). Furthermore, the most significantly enriched pathway in KEGG analysis was “tryptophan metabolism” in both G18 and P7 (Fig. 5E,F). In fact, the expression levels of *Tph1* and *Tph2*, which encode the 2 isoforms of tryptophan hydroxylase (the rate-limiting enzyme in the 5-HT synthesis), were significantly upregulated in the islets at G18 and P7 compared with nonpregnant islets, whereas their mRNA levels were robustly decreased in the islets at P1 compared with pregnant islets at G18 (Fig. 5G,H). There was no significant difference in the expression levels of serotonin receptors between the groups (Fig. S4). Thus, the genes associated with serotonin synthesis were dynamically enhanced after parturition between P1 and P7 as well as during pregnancy.

### Serotonin production is enhanced in lactating female mice

**Figure 6:**
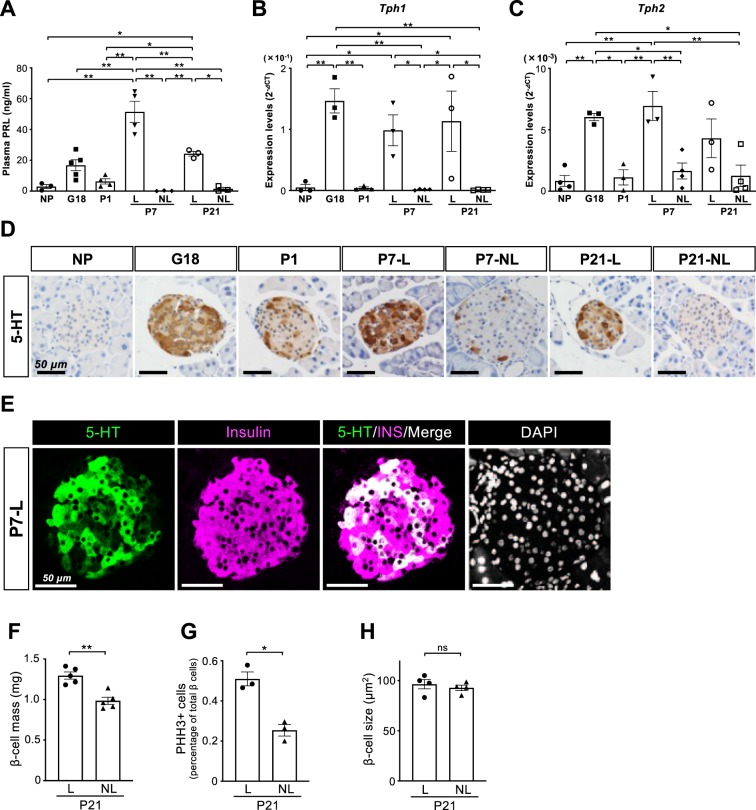
Serotonin production is enhanced in lactating female mice. (**A**) Plasma prolactin (PRL) levels were measured in the mice during perinatal period (n = 3–5). (**B**,**C**) Expression levels of *Tph1* and *Tph2* mRNAs in the islets of lactating and nonlactating mice were investigated by real-time RT-PCR. NP, non-pregnant female; G, gestational day; P, postpartum day; L, lactating females; NL, nonlactating females. *P < 0.05; **P < 0.01. (**D**) Immunostaining for 5-HT in the mouse pancreata at different perinatal stages. Scale bars, 50 μm. (**E**) Double immunostaining for 5-HT (green) and insulin (magenta) in the pancreas of a lactating mouse at P7. Scale bars, 50 μm. (**F–H**) Quantification of β-cell mass (**F**), percentage of β cells positive for PHH3 (**G**), and β-cell size (**H**) was performed in the pancreata of lactating and nonlactating mice (n = 3–5). *P < 0.05; **P < 0.01, and ns, not significant. Data are presented as the mean ± SE.

As the lactogenic hormone prolactin (PRL) has been reported to induce Tph1 expression in the islets and mammary gland^7,11^, we investigated whether increased 5-HT synthesis is associated with lactation after parturition. To address this point, plasma PRL levels and parameters of 5-HT synthesis were measured in pregnant and postpartum females, including postpartum lactating (L) mice and non-lactating (NL) mice at P7 and P21. As shown in Fig. 6A, plasma PRL levels demonstrated the highest peak in lactating P7 mice, and were also significantly higher in lactating P21 mice than nonpregnant and nonlactating females. To quantify the temporal changes in *Tph1* and *Tph2* mRNAs, real-time RT-PCR was performed on the islets of lactating and nonlactating mice, which demonstrated a significant increase in *Tph1* and *Tph2* levels in the islets of lactating mice at P7 compared with nonpregnant and nonlactating female mice (Fig. 6B,C). In addition, the expression level of *Tph1* was also significantly increased in the islets of lactating mice at P21 compared with the islets of nonpregnant mice. To directly assess serotonin synthesis in the islets, immunostaining for 5-HT was performed throughout the perinatal period with or without lactation (Fig. 6D). The results demonstrated that a substantial number of 5-HT positive cells were observed in the islets of postpartum lactating mice at P7 as well as in pregnant mice at G18, whereas only a few islet cells were positive for 5-HT in the islets of postpartum nonlactating mice at P7 and P21, consistent with the robust reduction of *Tph1* mRNAs in nonlactating mice. To further assess the effects of lactation on β-cell homeostasis, β-cell mass, cell size, and the number of PHH3-positive cells were compared between lactating and non-lactating female mice. Double immunostaining for 5-HT and insulin demonstrated that all 5-HT-positive cells were stained for insulin (Fig. 6E). As shown in Fig. 6F,G, β-cell mass and the number of PHH3-positive β-cells at P21 were significantly higher in lactating mice than in nonlactating mice, whereas there was no significant difference in β-cell size (Fig. 6H). Taken together, these data indicate that postpartum β-cell mass and proliferation are increased by lactation, which may be associated with enhanced serotonin production in the islets.

### Serotonin production in postpartum β cells in human

**Figure 7:**
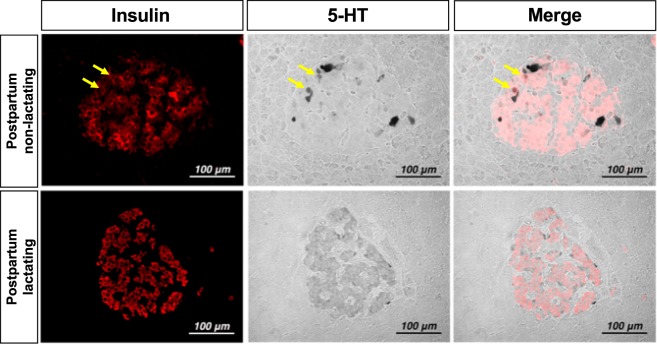
Immunohistochemical staining for 5-HT in autopsied pancreas of human subjects after parturition. Immunohistochemical costaining labels insulin (left, red fluorescence) and 5-HT (middle, black) in pancreata of human autopsy samples from postpartum day 0 (5 hours) without lactation (upper panels) and postpartum day 2 with lactation (lower panels).

To confirm whether serotonin production is detected in humans as well as in mice, double immunostaining for 5-HT and insulin was performed in human pancreatic tissues from 2 postpartum cases (Fig. 7). In the islets of a nonlactating postpartum subject, a small number of insulin-producing cells showed weak-to-moderate expression of 5-HT, whereas the cells that highly expressed 5-HT were negative for insulin, glucagon, and somatostatin (Fig. S5). In contrast, almost all insulin-producing cells moderately expressed 5-HT in the islets of a lactating postpartum subject.

## Discussion

Whereas the substantial increase in β-cell mass during pregnancy has been known for decades^1–3,5,7^, few studies have focused on the temporal dynamics of β-cell mass and the underlying cellular mechanisms after parturition. In this study, several lines of evidence demonstrate that the rapid reduction of β-cell mass after parturition is determined by reduced β-cell size and proliferation, rather than by increased apoptosis or cellular reprogramming to other cell types. In addition, our data clarified the possible mechanisms behind the dynamic changes in β-cell proliferation after parturition, which is reactivated several days after parturition, accompanied by enhanced serotonin synthesis during lactation.

Because we used five groups of pregnant or postpartum females that were sacrificed at approximately the same age (12–13 weeks), the timing of pregnancy differed among the groups (Fig. 1A), which should be taken into account in the interpretation of the findings. For example, the postpartum “P21” females became pregnant at the age of 6–7 weeks, which is 3–4 weeks younger than when the “P1” females became pregnant, and it is possible that pregnancy and parturition at an earlier age may affect β-cell mass and other parameters. To overcome these limitations, additional experiments should be performed using postpartum females that become pregnant at the same age and are sacrificed at the different postpartum stages.

Although a previous study reported the contribution of cellular apoptosis in the reduction of β-cell mass after parturition in the rat pancreas^4^, TUNEL staining in our study showed no significant increase in apoptotic cell number in the islets of postpartum mice (Fig. S3). In addition, our RNA-seq data demonstrated no significant increase in apoptosis-related genes, such as caspase 3 (*Casp3*), *Bax*, and *Bcl-2*, which have been reported to contribute to β-cell apoptosis^12,13^. Collectively, our data suggest that β-cell apoptosis is not the cause of reduced β-cell mass after parturition, at least in our *in vivo* experiments in mice.

We further performed lineage tracing experiments using *Ins1-Cre*; *Rosa26*^*lacZ*^ (*βLacZ*) reporter mice, which resulted in no evidence for the cellular reprogramming of β cells into non-β cells (Fig. 2). It was reported that *Foxo1*-deficient β cells can be converted into other cell types, such as progenitor-like cells and glucagon-expressing cells, after multiparity^9^, which implies that β-cell fate can be changed after parturition under certain specific conditions. Although further analysis at several postpartum stages is needed to clarify this issue, our data suggest that β-to-non-β transition is not likely to be a major cause of reduced β-cell mass after parturition.

The precise quantification of β-cell size (Figs. S1 and S2) demonstrated that the increased β-cell size at G18 was robustly decreased after parturition at P7, which was also significantly smaller than that of nonpregnant mice (Fig. 1D). Given the known role of ribosomal protein (RP) S6 kinase beta-1 (S6K1) in controlling pancreatic β-cell size^10^, we analyzed the expression levels of *S6K1* (*Rps6kb1*) mRNAs based on RNA-seq data, and found that the increased expression of *Rps6kb1* mRNAs at G18 was significantly decreased after parturition at P1 and P7 (Fig. 4L), which can at least partly explain the decreased β-cell size after parturition (Fig. 1D).

Direct RNA sequencing of isolated islets during the perinatal period demonstrated temporal dynamic changes in global expression patterns within only a day after parturition between G18 and P1 (Fig. 3A,B). Notably, positive regulators of cellular proliferation were dramatically downregulated in the islets at P1 compared with at G18 (Figs. 3C–E, and 4A–L). To our surprise, the expression levels of *Cdc25c*, *Cdc20*, *Ccnb1*, and *Ccnb2* in the islets at P1 were significantly decreased compared with the non-pregnant islets (Fig. 4), implying that parturition negatively regulates cellular proliferation below normal homeostatic levels. Collectively, such substantial changes in cell cycle-related mRNAs, together with reduced β-cell size, regulate the significant decrease in β-cell mass after parturition.

RNA sequencing and unbiased enrichment analyses using GO and KEGG annotations demonstrated the significant increase in the expression levels of serotonin metabolism-related genes in the islets of postpartum females at P7 as well as in pregnant mice (Fig. 5A–H). Serotonin has been reported to regulate β-cell expansion during pregnancy in both humans and mice, and serotonin production is induced by PRL and placental lactogen^7,14^. In addition, PRL signaling has been demonstrated to play an important role in β-cell expansion during pregnancy^3,15^. Thus, whereas many studies have reported the importance of serotonin and prolactin signaling in the regulation of β-cell mass during pregnancy, it still remains unclear as to how β-cell mass is regulated by serotonin and PRL after parturition. In this study, all these parameters were compared between lactating and nonlactating female mice, demonstrating that serotonin production was significantly enhanced in lactating females, which paralleled with β-cell proliferation (Fig. 6G). These findings strongly suggest that PRL and serotonin signaling play important roles in β-cell expansion, not only during pregnancy but also during lactation. As clinical studies have shown the protective effect of breastfeeding on the development of diabetes in humans^16–18^, β-cell proliferation enhanced by lactation may contribute to a lower risk of glucose intolerance. Further studies are needed to clarify how increased plasma PRL and 5-HT production improve β-cell function during lactation as well as during pregnancy. It is of interest and importance to further compare islet transcriptome changes between lactating and non-lactating mice at P21. Such data would provide us with a better understanding towards controlling β-cell mass to treat diabetes.

## Methods

### Animals

*C57BL/6 J* female mice were purchased from Sankyo Labo Service Corporation (Tokyo, Japan), and were sacrificed at the age of 12–13 weeks. Postpartum lactating females at P7 or P21 were used only when the mice breastfed more than 6 pups. Postpartum nonlactating females were prepared by separating their pups within a day after parturition. *Ins1-Cre* and *ROSA26*^*LacZ*^ reporter mice (R26R) were generated as described previously^19,20^. To trace β-cell fate, *Ins1-Cre* mice were crossed with *R26R* mice to generate *Ins1-Cre; R26R* double-mutant mice. Mice were housed on a 12-h light/dark cycle in a controlled climate and fed standard rodent food.

### Clinical history of the human pancreas samples

Human pancreas specimens were obtained at autopsy from the archives of the Department of Pathology and Molecular Medicine, Hirosaki University Graduate School of Medicine. All specimens were fixed with neutral buffered formalin and embedded in paraffin. The postpartum nonlactating subject was a 35-year-old woman who died 5 hours after delivery owing to pulmonary embolism of amniotic fluid at 39 weeks of pregnancy (Fig. 7). The postpartum lactating subject was a 25-year-old woman who died of a dissecting aneurism 2 days after delivery (Fig. 7).

### Immunohistochemical analysis of mouse pancreata

Tissues were fixed in 4% paraformaldehyde in phosphate-buffered saline (PBS) at 4 °C, washed in PBS, immersed in sucrose solution, and embedded in Tissue-Tek (OCT Compound, Sakura, Japan), or processed routinely for paraffin embedding. The primary antibodies used in this study were the following: guinea pig anti-insulin (1:5; Dako, Carpinteria, CA), rat anti-insulin (1:200; R&D Systems), rabbit anti-glucagon (1:1000; Dako, Carpinteria, CA), guinea pig anti-glucagon (1:1000; TAKARA BIO, Otsu, Japan), rabbit anti-PHH3 (Ser10) (1:200; Cell Signaling, Danvers, MA), rat anti-E-cadherin (1:400; Abcam, Cambridge, MA), rabbit anti-serotonin (1:2000; Immunostar, Hudson, WI), and rabbit anti-β-galactosidase (1:200; MBL, Nagoya, Japan). For the detection of PHH3 and E-cadherin, mounted sections were microwaved at 95 °C for 20 min in citrate buffer (pH 6.0) for antigen retrieval. Apoptotic cells were detected by TUNEL staining using *in situ* Apoptosis Detection Kit (TAKARA BIO, Japan). The secondary antibodies used in this study were Alexa Fluor 488-conjugated anti-guinea pig IgG, Alexa Fluor 488-conjugated anti-rabbit IgG, Alexa Fluor 555-conjugated anti-guinea pig IgG, Alexa Fluor 555-conjugated anti-rabbit IgG, Alexa Fluor 594-conjugated anti-rat IgG, Alexa Fluor 633-conjugated anti-guinea pig IgG, Alexa Fluor 633-conjugated anti-rabbit IgG, and Alexa Fluor 633-conjugated anti-rat IgG (all at 1:200; Invitrogen, Carlsbad, CA). For the detection of serotonin, sections were incubated with secondary rabbit anti-biotin (1:300; Dako, Carpinteria, CA) for 30 min at room temperature and then incubated with peroxidase-conjugated streptavidin (1:300; Dako) for 30 min at room temperature for signal amplification. Immunoreactivity was visualized with 3,3′-diaminobenzidine (DAB, Dako) before brief counterstaining with hematoxylin. The slides were dehydrated and mounted. Slides were imaged using a Leica TCS SP5 confocal laser-scanning microscope (Wetzlar, Germany).

For measuring β-cell mass, five different sections at least 1,200 μm apart in total were selected and stained with an anti-insulin antibody. The ratio of β-cell area/whole pancreas area (relative β-cell area) was digitally quantified using BZ Analyzer (Keyence, Osaka, Japan). The β-cell mass was calculated by multiplying relative β-cell area by pancreatic weight. For evaluating β-cell size and proliferation, more than 2,000 β cells were quantified in more than three mice per group. The number of TUNEL-positive cells was counted in more than 50 islets from three mice per group.

### Measurement of plasma PRL levels

Heparinized blood samples were centrifuged, and the supernatant was collected and stored at −80 °C. Plasma prolactin levels were measured using ELISA kits according to the manufacturers’ protocols (Abcam, Cambridge, MA).

### Real-time quantitative PCR

The islets were isolated from 12 to 13-week old *C57BL/6 J* female mice at different stages of the perinatal period (NP, G18, P1, P7, P21; *n* = 3–4 mice per group). Total RNA was extracted from the isolated islets using RNeasy Plus Mini Kit (Qiagen, Valencia, CA). Extracted total RNAs were linearly amplified and converted into cDNA with NuGEN RNA Amplification System (NuGEN, San Carlos, CA), and individual cDNAs were quantified by real-time PCR using TaqMan primer-probe sets (Applied Biosystems, Foster City, CA). Gene expression levels of the assayed genes were normalized to the expression levels of *glucuronidase beta* (*Gusb*, Mm01197698_m1).

### RNA-sequencing

Islets were isolated from 12-week-old *C57BL/6 J* female mice (*n* = 5–6 mice per group) at different stages of the perinatal period (NP, G18, P1, and P7). Total RNA was extracted using RNeasy Plus Mini Kit (Qiagen). RNA quality was tested using the Agilent 2100 BioAnalyzer (Agilent Technologies, Palo Alto, CA), and cDNA preamplification was performed using the SMART-Seq v4 Ultra Low Input RNA Kit for Sequencing (Clontech, Mountain View, CA). The sequencing libraries were prepared using the Nextera XT DNA Library Prep Kit (Illumina, San Diego, CA). All samples had a peak size of approximately 500 base pairs and were sequenced on the HiSeq 2500 platform (Illumina).

Sequence reads were mapped against the Mus musculus genome assembly (Genome Reference Consortium GRCm38, UCSC version mm10). MultiExperiment Viewer (Mev) version 4.8.1 was used for hierarchical clustering and heatmaps. The Gene Ontology and pathway enrichment analysis were performed using DAVID version 6.8.

### Immunostaining for 5-HT in the human pancreas

Immunohistochmesirty for 5-HT was first performed with a biotin-free polymer detection system for 5-HT (Biocare Medical LCC, CA). Briefly, 4-μm thick deparaffinized sections were immersed in Tris-buffered saline (TBS) and subsequently placed in a pressure chamber (Pascal, DAKO Cytomation, CA) for antigen retrieval at 125 °C for 20 minutes in Tris-EDTA buffer. Endogenous peroxidase was blocked in 3% hydrogenperoxide solution. Then the sections were incubated with a primary antibody against 5-HT (1:1,000, rabbit polyclonal, Immunostar Inc., WI) overnight at 4 °C. After the application of the secondary antibody for rabbit IgG, the immunoreaction products were colorized with diaminobenzidine (Thermo Fisher Scientific, MA). Subsequently, immunofluorescence analysis for insulin was performed. Sections were incubated with the primary antibodies against Insulin (1:250, guinea pig polyclonal, BIORAD, CA), glucagon (1:1,000, mouse monoclonal, Abcam, Cambridge, MA), and somatostatin (1:500, goat polyclonal, Santa Cruz Biotechnology, CA) for overnight at 4 °C followed by incubation with Alexa-fluor 594-conjugated secondary antibodies (1:500, Thermo Fisher Scientific, MA) for 2 hours at room temperature. Specificity was confirmed by the lack of positive staining after omission of the first antibody or replacement with nonimmune serum. Immunofluorescence sections were observed by fluorescence microscopy (Axio imager M1, Carl Zeiss, Germany).

### Statistical analysis

Statistical analyses were performed using the SPSS 18.0 for Windows (SPSS Inc., Chicago, IL). The Student *t*-test was performed to determine associations between lactation and non-lactation groups. Multiple groups were analyzed by one-way ANOVA with a multiple comparison test, followed by the *post hoc* Tukey’s test. A *P*-value of less than 0.05 was considered to indicate a statistically significant difference between 2 groups. All data are presented as the mean ± SE.

### Study approval

The study protocol was reviewed and approved by the Animal Care and Use Committee of Juntendo University, and all the experiments were performed in accordance with the relevant guidelines and regulations.

Human pancreatic specimens were obtained at autopsy with the informed consent of the legal next-of-kin in accordance with the guidelines of the Hirosaki University Institutional Review Board. The study design was approved by the ethics committee of the Hirosaki University School of Medicine (approval number 2014-269), and the study conforms to the provision of the Declaration of Helsinki.

## Supplementary information

Figure S1-S5.
