# Supplementary material for: Biphasic changes in β-cell mass around parturition are accompanied by increased serotonin production
Source: Sci Rep. 2020 Mar 18;10:4962. doi: 10.1038/s41598-020-61850-1 (PMC7080838; doi:10.1038/s41598-020-61850-1)
Supplement: Supplementary file 1 — Figure S1-S5. [file 41598_2020_61850_MOESM1_ESM.pdf]

Biphasic changes in β-cell mass around parturition are accompanied by increased serotonin production

Masaya Takahashi1, Takeshi Miyatsuka, Luka Suzuki, Sho Osonoi,

Miwa Himuro, Masaki Miura, Takehiro Katahira, Yuka Wakabayashi,

Ayako Fukunaka, Yuya Nishida1, Yoshio Fujitani, Satoru Takeda,

Hiroki Mizukami, Atsuo Itakura, and Hirotaka Watada

Supplementary figures S1−S5

Figure S1.

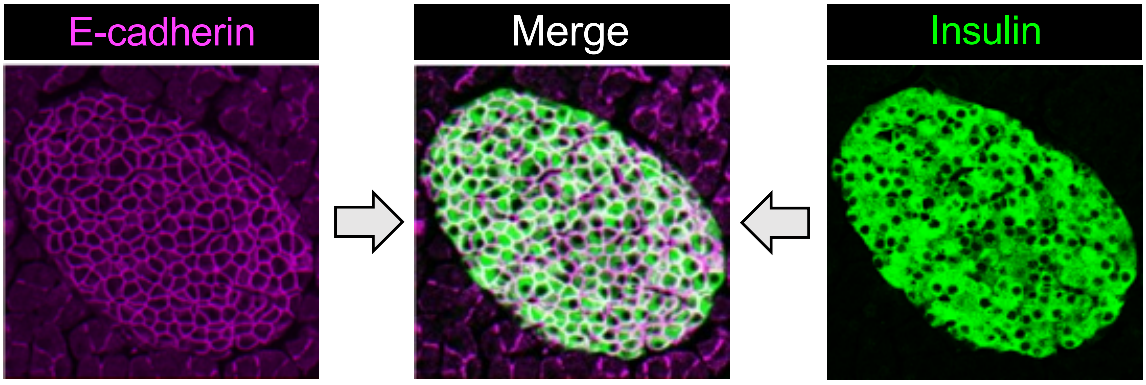

$$\text{Mean individual } \beta\text{-cell size } (\mu m^2) = \frac{\text{Total } \beta\text{-cell area}}{\text{The number of } \beta \text{ cells}}$$

Figure S1. Measurement of mean β-cell size.
Schematic diagram demonstrating the analysis of mean β-cell size. Through immunostaining for insulin (green) and E-cadherin (magenta), total β-cell area and the number of β cells were measured. The mean β-cell size was calculated by dividing the total β-cell area by the number of β cells.

Figure S2.

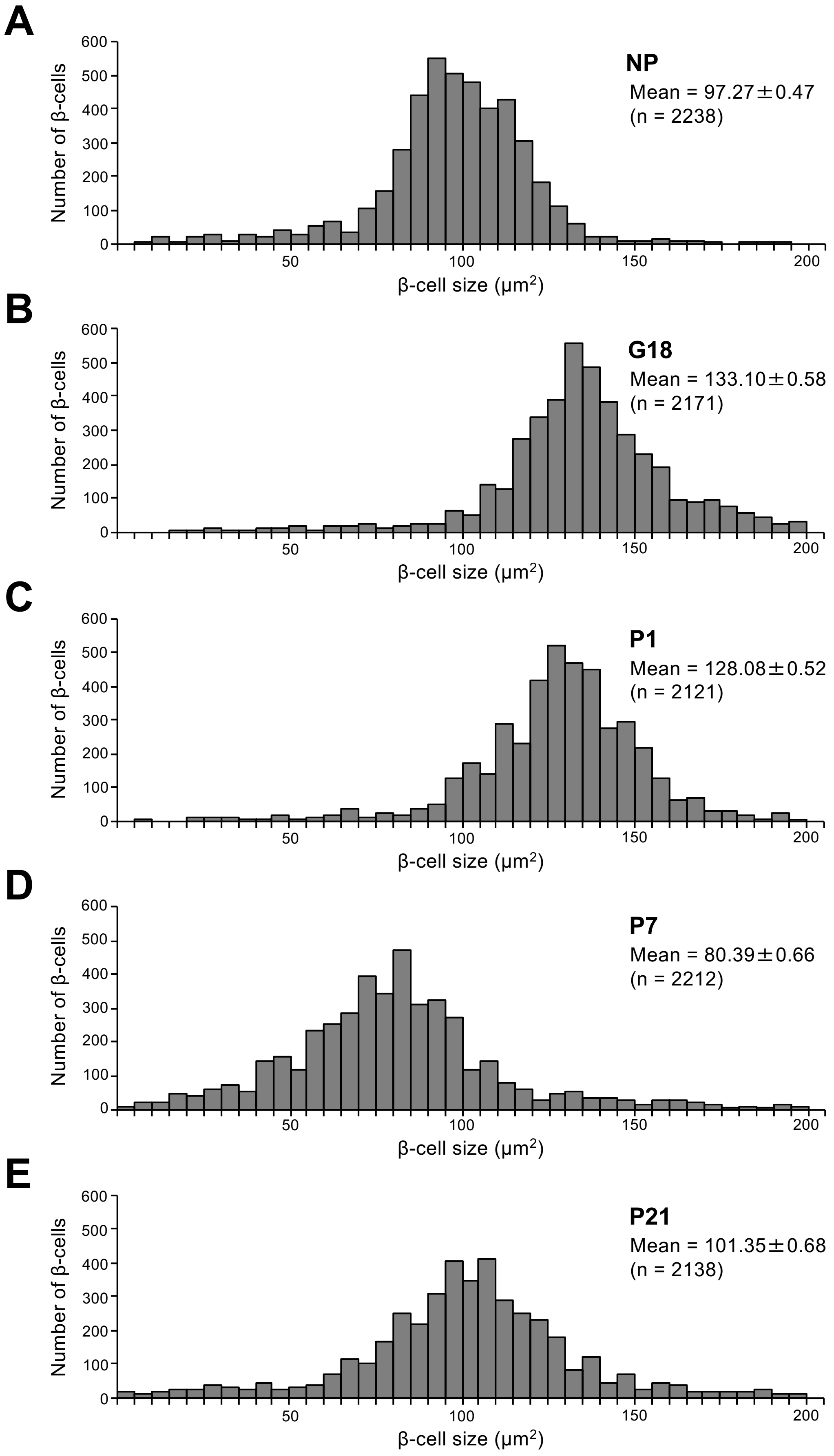

Figure S2. Heterogeneity of β-cell size around parturition.
Individual β-cell sizes were measured by immunostaining for insulin and E-cadherin (as shown in Figure S1), and histograms were created for all groups. NP, non-pregnant female; G, gestational day; P, postpartum day. Lactating mice were used for measuring individual β-cell sizes at P7 and P21.

Figure S3.

A

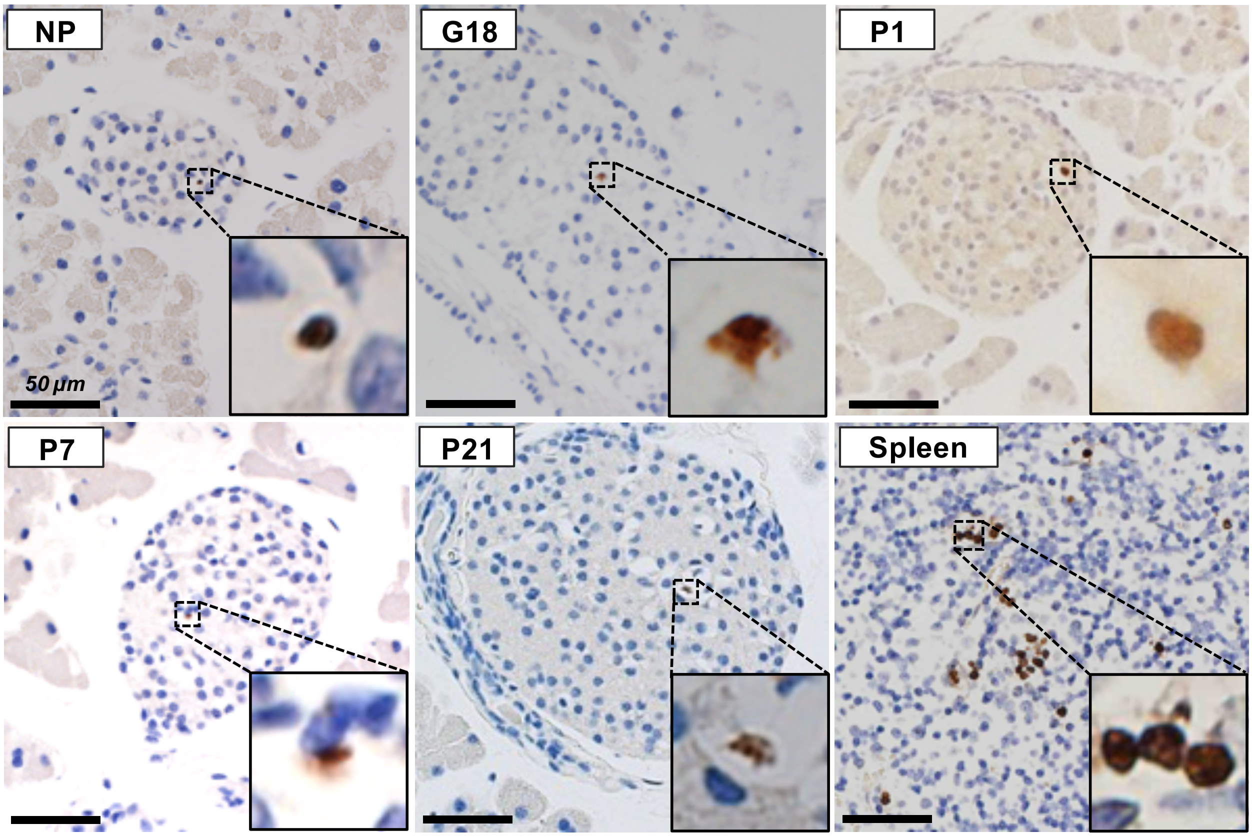

B

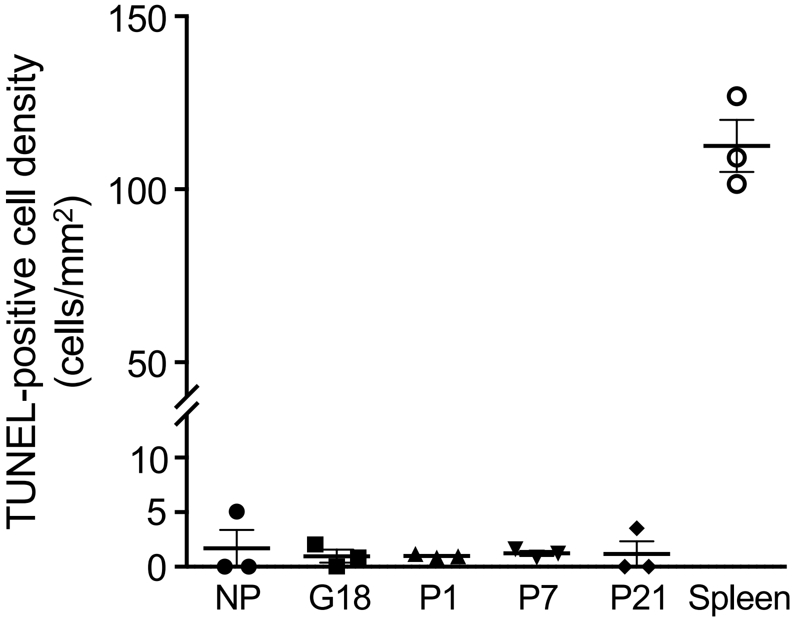

Figure S3. No significant changes in islet apoptosis during the perinatal period. (A) The number of apoptotic cells in the islets was quantified by TUNEL staining in 12–13-week-old C57BL/6J female mice at different perinatal stages (NP, G18, P1, P7, and P21). For the samples of postpartum mice at P7 and P21, only lactating mice were used. The spleen was used as a positive control. Scale bars, 50 μm. (B) The number of TUNEL-positive cells in each islet was counted and normalized to the islet areas (n = 3). NP, nonpregnant female; G, gestational day; P, postpartum day.

Figure S4.

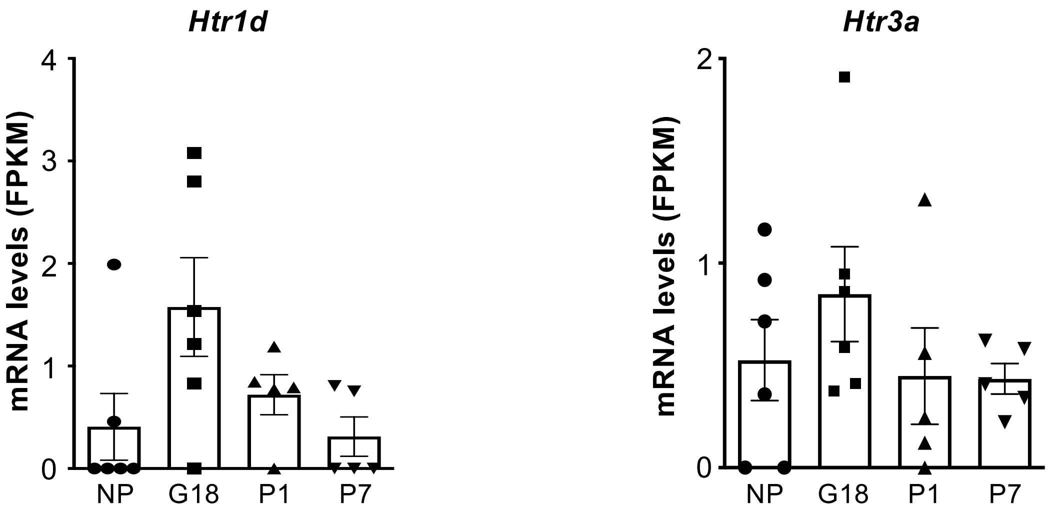

Figure S4. Dynamic changes in expression levels of serotonin receptors during the perinatal period.
Expression levels (FPKM) of Htr1d and Htr3a are shown as bar graphs with dots indicating individual data (n = 5–6 mice), based on RNA-sequencing results. NP, nonpregnant females; G, gestational day; P, postpartum day. Data are presented as the mean ± SE.

Figure S5.

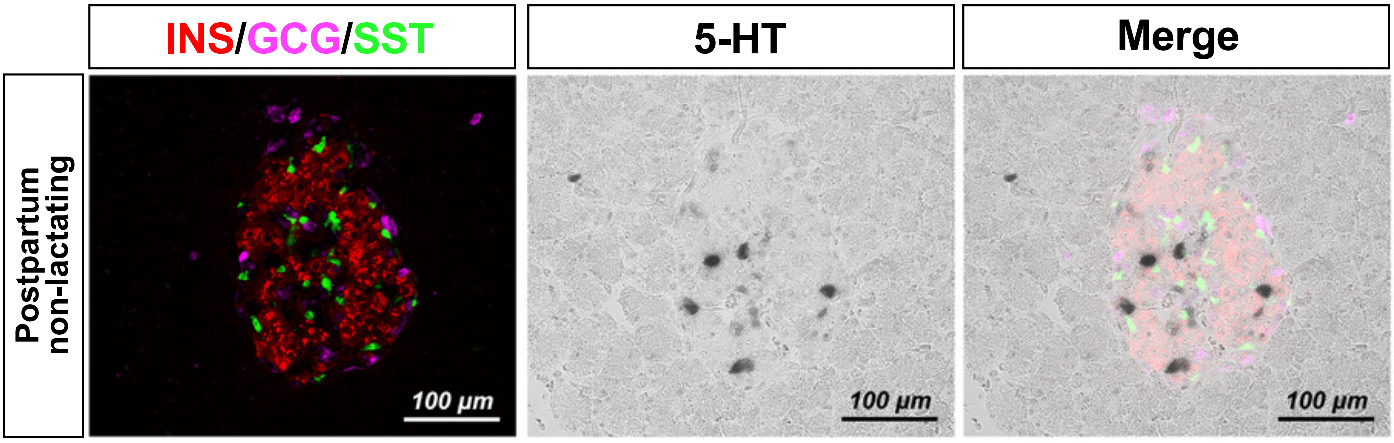

Figure S5. Immunohistochemical staining for 5-HT and pancreatic endocrine hormones in an autopsied pancreas from a nonlactating human subjects after parturition.
Immunohistochemical costaining labeled insulin (INS, red in the left panel), glucagon (GCG, magenta in the left panel), somatostatin (SST, green in the left panel), and 5-HT (black in the middle panel) in the pancreas of a human autopsy sample from postpartum day 0 (5 hours) without lactation.
